# Staging practices and breast cancer stage among population-based registries in the MENA region

**DOI:** 10.1016/j.canep.2022.102250

**Published:** 2022-12

**Authors:** Marion Piñeros, Ophira Ginsburg, Karima Bendahhou, Sultan Eser, Wael A. Shelpai, Heba Fouad, Ariana Znaor, Staging Survey Group, Doudja Hammouda, Sabiha Bouzbid, Fayçal Beichi, Khaoula Bouharati, Fadhila Toudeft, Nabiel Nazmi Hanna Mikhail, Khitam Mohseen Ali Al-Aubaidy, Omar Nimri, Eman Janahi, Amani Elbasmi, Nada Ghosn, Adel A. Attia, Waled Masaud, Mohammed Adnane Tazi, Huda Lahham, Elias Mamo Alemayehu, Mona Numairi, Najla Al Lawati, Hyem Khiari, Hülya Karakilinç, Cankut Yakut

**Affiliations:** a Alger Cancer Registry, Algeria; b Annaba Cancer Registry, Algeria; c Batna Cancer Registry, Algeria; d Setif Cancer Registry, Algeria; e Tizi-Ouzu Cancer Registry, Algeria; f Egypt National Cancer Registry, Egypt; g Iraq National Cancer Registry, Iraq; h Jordan National Cancer Registry, Jordan; i Bahrain National Cancer Registry, Bahrain; j Kuwait National Cancer Registry, Kuwait; k National Cancer Registry, Lebanon; l Benghazi Cancer Registry, Libya; m Libya National Cancer Registry, Libya; n Rabat Cancer Registry, Morocco; o Palestine; p National Cancer Registry, Qatar; q National Cancer Registry, Sudan; r National Cancer Registry, Oman; s North Tunisia Cancer Registry, Tunisia; t Antalya Cancer Registry, Turkey; u Izmir Provincial Cancer Registry, Turkey; v Cancer Surveillance Branch, International Agency for Research on Cancer, Lyon, France; w Center for Global Health, US National Cancer Institute, MD, USA; x Casablanca Cancer Registry, Casablanca, Morocco; y Balıkesir University, Faculty of Medicine, Balikesir, Turkey; z National Cancer Registry, Dubai, United Arab Emirates; aa WHO Regional Office for the Eastern Mediterranean, Cairo, Egypt

**Keywords:** Cancer, Surveillance TNM staging, Northern Africa, Middle East, Early detection

## Abstract

**Background:**

Availability of stage information by population-based cancer registries (PBCR) remains scarce for diverse reasons. Nevertheless, stage is critical cancer control information particularly for cancers amenable to early detection. In the framework of the Global Initiative for Cancer Registry Development (GICR), we present the status of stage data collection and dissemination among registries in the Middle East and Northern Africa (MENA) region as well as the stage distribution of breast cancer patients.

**Methods:**

A web-based survey exploring staging practices and breast cancer stage was developed and sent to 30 PBCR in 18 countries of the MENA region.

**Results:**

Among 23 respondent PBCR, 21 collected stage data, the majority (80%) for all cancers. Fourteen registries used a single classification (9 TNM and 5 SEER), 7 used both staging systems in parallel. Out of 12,888 breast cancer patients (seven registries) 27.7% had unknown TNM stage (11.1% in Oman, 46% in Annaba). When considering only cases with known stage, 65.3% were early cancers (TNM I+II), ranging from 57.9% in Oman to 83.3% in Batna (Algeria), and 9.9% were stage IV cancers. Among the nine registries providing SEER Summary stage for breast cancer cases, stage was unknown in 19% of the cases, (0 in Bahrain, 39% in Kuwait). Stage data were largely absent from the published registry reports.

**Conclusion:**

Despite wide stage data collection by cancer registries, missing information and low dissemination clearly limit informing efforts on early detection. The use of two classification systems in parallel implies additional workload and might undermine completeness. The favourable results of early cancer (TNM I+II) in two thirds of breast cancer patients needs to be interpreted with caution and followed up in time. Although efforts to improve quality of stage data are needed, our findings are particularly relevant to the WHO Global Breast Cancer Initiative.

## Highlights

• Stage data are widely collected by population-based cancer registries (PBCR) in 16 MENA countries.
• Stage data were scarcely included in the registry reports.
• Both TNM and SEER classifications are used by PBCR in this region.
• Amongst 13,000 breast cancer patients across 23 PBCR, slightly more than 65% were diagnosed at early stages (TNM I, II).
• The quality and dissemination of stage information needs improvement to better inform cancer control.

## Nomenclature

EMRO: Regional Office for Eastern Mediterranean of the World Health Organization
GICR: Global Initiative for Cancer Registry Development
GBCI: Global Breast Cancer Initiative
IARC: International Agency for Research on Cancer
LMIC: Low- and Middle-Income Countries
MENA: Middle East and Northern Africa
PBCR: Population-based cancer registries
SEER: Surveillance, Epidemiology, and End Results (SEER) Program
TNM: Tumour, Node and Metastasis staging system
WHO: World Health Organization
UICC: Union for International Cancer Control

## 1 Introduction

Establishing the extent of disease at the time of diagnosis is a prerequisite for evidence-based decision making in cancer management. In the public health setting, information on stage of disease -provided by population-based cancer registries (PBCR)- permits a better understanding of the burden, gives an indication of the awareness and access to cancer diagnosis and care, and is necessary to evaluate early detection activities in the population [1]. In addition, stage at diagnosis is a key component in population-based studies on cancer survival. In clinical research, stage information may determine the eligibility for specific interventions in clinical trials.

While the TNM classification is the widely-used standard to classify stage of disease in the clinical setting [2], [3], among population-based cancer registries stage data collection involves different challenges that affect quality, and not all collect information on TNM stage. PBCRs tend to use classification systems specifically developed for registries, such as the SEER Staging system [4] or using both TNM and SEER system in parallel, resulting in poor comparability of PBCR data globally [5].

To improve availability of staging information, particularly in less resourced settings, Essential TNM, a complementary TNM system to be used by cancer registries when complete TNM elements are not available was developed some years ago, focusing initially on the main cancer sites amenable to early detection [5].

As part of the Global Initiative for Cancer Registry Development (GICR) activities in the Regional Hub for Northern Africa, Central Asia and Northern Africa, a virtual course on TNM and Essential TNM staging took place in November 2020 for participants from countries in the Middle East and Northern Africa (MENA) region (as defined in the Methods section). The training was followed up by a survey to assess current staging practices; in addition, we collected stage data for breast cancer, as a major cause of cancer morbidity, responsible for one third of cancer cases among women in this region [6].

Hereby, we document the staging practices in PBCR in MENA countries and establish availability of staging information with the aim to identify gaps that may help to improve the quality and use of stage data in the region. In addition, in selected PBCR, we compare the TNM stage distribution at diagnosis of breast cancer patients evaluating also the completeness of information. Findings are interpreted in the framework of the WHO Global Breast Cancer Initiative, that aims, amongst others, for achieving 60% or more of cases detected at early stages (TNM I-II) [7].

## 2 Materials and methods

We developed a descriptive study, addressing a questionnaire to population-based cancer registries in the MENA region and requesting them also to provide staging data for breast cancer.

The questionnaire was developed in English, structured in four parts as follows:

Part 1 contained basic general information on the PBCR. In Part 2 we asked about stage data collection in the PBCR, the classification system used, the cancer sites (all or selected sites) for which stage data are collected; knowledge and use of Essential TNM; and whether data abstraction from clinical records was predominantly paper-based or electronically based. We asked explicitly for TNM and SEER Summary Stage, without reference to a specific edition. Part 3 was exclusively for TNM users exploring whether they collected pTNM, cTNM, or combined information, for which specific sites, the time since use, and the main challenges associated with TNM data collection. Finally, Part 4 addressed only registries not using TNM, asking for the reasons and their potential interest in collecting it. Part 3 and 4 included open-ended questions to explore the challenges and perceived barriers as well as suggestions for improving the use and completeness of data. No pilot questionnaire was used considering the few questions and the knowledgeable personnel to whom they were addressed.

In partnership with the GICR Regional Hub for Northern Africa, Central and Western Asia we defined MENA countries/territories as: Algeria, Bahrain, Djibouti, Egypt, Iraq, Jordan, Kuwait, Lebanon, Libya, Morocco, Oman, Palestine, Qatar, Saudi Arabia, Somalia, Sudan, Syria, Tunisia, the United Arab Emirates, Turkey and Yemen. Operational population-based cancer registries were selected based on the information collected within the GICR programme and the WHO Regional Office for Eastern Mediterranean (WHO EMRO) [8]. Thus, the questionnaires were sent to 30 national or subnational PBCR in 18 countries/territories. These also included the participants of the 2020 Staging Course from Algeria, Iraq, Jordan, Kuwait, Lebanon, Oman, Saudi Arabia, Tunisia, Turkey, West Bank and Gaza Strip, and the United Arab Emirates.

The survey was designed in Redcap hosted at IARC [9], [10]; answers and information on latest report were collected between May and August 2021.

In addition to the questionnaire, the participants were asked to provide stage data for breast cancer for the period 2013–2017 or the latest period available, as well as the most recent cancer registry report published. In the stage data, we did not differentiate between unknown and missing for the “unknown stage” and refer to unknown along the text. Responses were exported into Microsoft Excel, used also to prepare the figures. For the purpose of comparability, in addition to overall stage distribution, we presented the data for known stages only.

For registries that did not send their latest registry report, we consulted the registry website, when available.

## 3 Results

We received responses from 23 PBCR in 16 countries out of which 12 had national coverage (Table 1).

**Table 1:** Availability of stage information and staging system used by population-based cancer registries in the MENA region.

Country/ Region	Population covered	Availability Stage	Class. System	Year of last report	Period reported	Stage in report - Class. System
Algeria, Alger	2,988,145 (2008, Census )	All sites	SEER	2020	2018	No
Algeria, Annaba	609,499 (2008, Census )	Selected sites	TNM	2021	2017-2019	NA
Algeria, Batna	1,119,791 (2008, Census )	Selected sites	TNM^&^	2019	2016	No
Algeria, Setif	1,489,979 (2008, Census )	Selected sites	TNM			
Algeria, Tizi-Ouzou	1,172,184 (2008, Census )	All sites	SEER	2019	2017	No
Egypt, National	104,613,288 (2021, UN)	All sites	SEER	2019	2012-2014	SEER
Iraq, National	40,222,493 (2021, UN)	All sites	SEER	2020	2019	No
Jordan, National	10,554,000 (2019)	All sites	TNM, SEER	2020	2017	No
Bahrain, National	1,771,190 (2021, UN)	All sites	TNM, SEER	2019	1998-2017	No
Kuwait, National	4,345,371 (2021, UN)	All sites	TNM, SEER	2021	2016	SEER
Lebanon, National	6,789,143 (2021, UN)	No	NA			
Libya, Benghazi	1,500,000 (2006, Census)	Selected sites	TNM	2015*	2003-2005	No
Libya, National	6,981,977 (2021, UN)	All sites	TNM	NA	NA	NA
Morocco, Rabat	1,907,071 (2021, UN)	All sites	TNM			
Morroco, Casablanca (Region)	4,723,840 (2020, HCP)	Selected sites	TNM			
Palestine, National	5,241,636 (2021, UN)	All sites	TNM, SEER	2021	2020	No
Qatar, National	2,938,651 (2021, UN)	All sites	TNM, SEER	2021	2017-2019	TNM
Sudan, National	45,059,810 (2021,UN)	No	NA			
Oman, National	5,260,127 (2021, UN)	All sites	TNM	2021	2018	TNM
Tunisia, North Tunisia	5,347,531 (2014, )	All sites	TNM	2021	2010-2014	SEER
Turkey, Izmir	4,321,000 (2019, Eurostat)	All sites	TNM^, SEER			
Turkey, Antalya	2,426,000 (2019, Eurostat)	All sites	SEER			
United Arab Emirates, National	9,282,410 (2020, Census)	All sites	TNM, SEER	2021	2017	SEER

& Stage data only for special studies; * Scientific article; ^Condensed TNM

UAE Census data: https://fcsc.gov.ae/en-us/Pages/Statistics/Statistics-by-Subject.aspx#/%3Ffolder=Demography%20and%20Social/Population/Population&subject=Demography%20and%20Social

Casablanca: https://www.hcp.ma/Les-projections-de-la-population-et-des-menages-entre-2014-et-2050_a1920.html

### 3.1 Stage data collection, classification system used and reporting of stage

Out of 23 registries, 21 collected stage data, 16 (80%) for all cancer sites and the remaining five registries for selected sites, all including breast, cervix, colorectal and prostate cancers.

Among those collecting stage data, 14 used a single classification system (9 used TNM and 5 used SEER), while the rest used the two staging systems in parallel. Overall, 15 registries used standard TNM Classification, one used Condensed TNM and 11 used SEER (Table 1). Only one registry mentioned using FIGO classification for gynaecological cancers in addition to TNM. Of the 15 registries using TNM, four answered that, if available, they collected both pTNM and cTNM.

The majority (18) of registries reported that in their data sources the clinical records were mainly paper-based; in contrast, Bahrain, Izmir (Turkey), Lebanon, Oman and Qatar reported that in their main sources, clinical records were mostly electronically- based.

Cancer registry reports were available for 15 PBCR (received either with the survey or consulted via a webpage). All of them, except one, were published between 2019 and 2021. For eleven registries, the time span between the date of the report and the data included was between 2 or 3 years; however, in four registries a considerable delay was observed (Table 1). Out of 13 of the registry reports by PBCRs that collect data on stage at diagnosis only six contained this information (Table 1).

### 3.2 Challenges associated with TNM data collection, solutions to improve

Nine registries addressed the question on the main challenges associated to recording TNM, which for the majority (seven) was the missing or incomplete TNM stage information in medical records. Other challenges brought up were the use of different staging systems in different data sources, and at the PBCR level, the lack of training and the workload that stage data collection entails.

Within the solutions proposed to improve TNM staging, two registries suggested more training (for both registrars and health professionals), while other two mentioned the necessity of providing regular feedback to hospitals to increase awareness about the importance of complete information. At the governance level, one registry suggested to unify and mandate the use of only one staging system in different health institutions within a country.

Knowledge about Essential TNM was high (10/13). Though none of the registries were current users, all expressed their interest in using it. Notably, all five Algerian respondent registries were interested in using it.

### 3.3 Stage at diagnosis among breast cancer patients in selected registries

We received breast cancer stage data from 17 registries, eight of them reporting in TNM and 10 in SEER; Bahrain reported in both systems. The TNM stage data from Qatar were excluded from the analysis as 80% were reported as unknown.

**Fig. 1:**
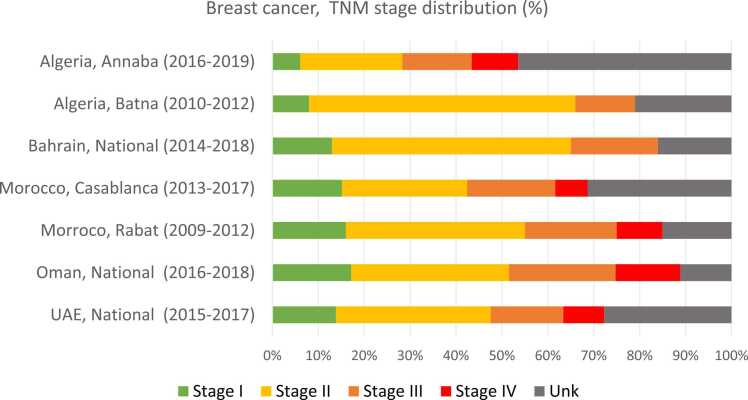
Stage at diagnosis (TNM classification; % distribution) among breast cancer patients, selected PBCR in the MENA region.

Fig. 1 shows the TNM stage distribution among 12,888 breast cancer patients in cancer registries of five countries, namely: Algeria (Annaba and Batna), Bahrain, Morocco (Casablanca and Rabat), Oman, and United Arab Emirates (UAE). Overall, 47.3% of the patients were diagnosed at early stages (13.4% at Stage I, 33.9% at Stage II), 17.9% at Stage III and 7.2% were stage IV cancers; TNM stage was unknown or missing in 27.7%, ranging from 11.1% in Oman to 46% in Annaba.

**Fig. 2:**
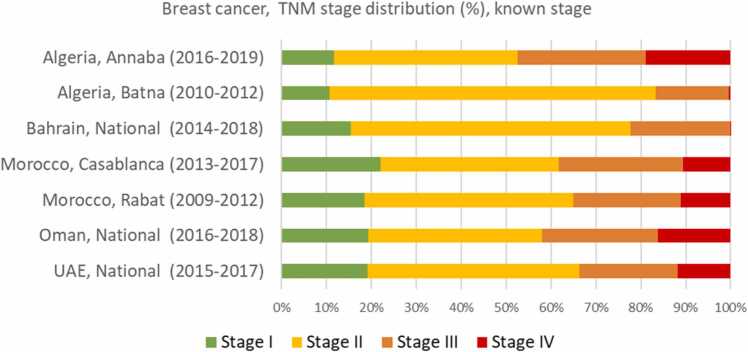
Stage at diagnosis (TNM classification; % distribution) among breast cancer patients with known stage, selected PBCR in the MENA region.

Fig. 2 illustrates the stage distribution among the 9 322 breast cancer patients with known TNM stage. Early-stage disease accounted for 65.4% ranging from 57.9% in Oman to 83.3% in Batna (Algeria) while 9.9% of the women were diagnosed with distant metastatic disease. Notably, in both, Bahrain and Batna (Algeria), less than 0.5% of the women were diagnosed with distant metastatic disease.

Ten registries provided the distribution of stage among breast cancer patients, using SEER Summary stage: Alger (Algeria), Tizi-Ouzu (Algeria), Bahrain, Iraq, Jordan, Kuwait, Palestine, Qatar, Antalya (Turkey) and Izmir (Turkey). Patients diagnosed with a localised cancer ranged from 14.1% in Antalya to 67% in Bahrain, while distant disease ranged from 11% (in four registries) to 20% in Kuwait. Proportions of cases with unknown stage had great variations, with lowest percentages in Bahrain and Palestine (0.5% and 6%) and highest with proportions of 30% or above in Jordan, Iraq and Kuwait respectively (Figure S1).

## 4 Discussion

Our results indicate that in the MENA region, stage information is widely recorded among PBCRs, predominantly for all cancer sites, and using both TNM and SEER Summary staging system. Despite being widely recorded, stage was not routinely included in half of the cancer registry reports, being available only upon request. The stage distribution among breast cancer patients varied among seven PBCR, with an overall 24% missing information. Among patients with known stage, two thirds of them were diagnosed at early stages (TNM I-II).

The collection of stage data for all cancer sites and the use of different classification systems among registries is common also to other regions and poses well-known difficulties for comparability and benchmarking data, particularly in international studies [5], [11]. We also found within country variability in staging practices in Algeria, a country that has full coverage by sub-national PBCR [12]. Furthermore, some registries in MENA use multiple stage classification systems simultaneously, a practice that imposes additional workload for registry personnel, particularly in a region where staff shortages and high turnover are common [13].

As stage classifications developed for use among cancer registries are not used by the clinical community, recommendations have been issued to record TNM as a unified system, understandable by both clinicians and public health specialists [11], [14]. If possible, registries should also document whether stage data are of clinical or pathological origin [14], which in our study was reported only by a minority of registries. Incomplete information in medical records was indicated as a major constraint, a concern already addressed previously and in other LMIC [15]. Even in high income settings while stage information might be present in the medical record, the TNM components are not often documented [16], [17].

We found that, despite variations between registries, overall, the number of breast cancer cases with unknown TNM stage represented one in every four cases. Though, as mentioned above, the high percentage of missing information could be mainly attributed to incomplete information in the clinical records or to their unavailability in the information source, it also warrants further exploration. For example, in Kuwait, the increasing number of patients that would be receiving a first treatment outside the country, has been indicated as a potential explanation to the worsening completeness of stage information in the registry in the last years [18]. This could also be the case in other of the Gulf countries with limited universal health coverage in the region [19], [20].

Incomplete information could in addition be contributing to the scarce dissemination of staging data by registries that we found. This clearly hinders the potential of registries for informing and monitoring public health programmes. Scarce visibility of PBCR data and lack of communication to policy makers was already identified in a previous survey [13]. Provision of feed-back to the oncology clinical community is also key, underlining their importance as primary source of the information while simultaneously aiming to improve completeness and quality of stage information. In MENA, the predominant location of PBCR in cancer and university hospitals, specifically in Northern Africa, is favourable for implementing specific strategies and establishing regular communication channels with clinicians. In turn, registries in governmental settings are in a better position to use stage data for planning, monitoring and evaluating national cancer control plans.

The reporting of cases of unknown stage is not always the case as seen in a systematic review on breast cancer stage [21] while being a practice that could avoid potential misinterpretations.

In 2020, WHO launched the Global Breast Cancer Initiative (GBCI), a collaboration to strengthen breast cancer control. A key target of the initiative is for all countries to achieve a minimum of 60% of breast cancers diagnosed at early stages [7]. In our study, this threshold was already attained for 9 300 breast cancer patients with known TNM stage. Our results are similar to the recently reported population- based data from mainly middle-income countries of the former soviet Union [22], but far more favourable than the results reported by ten PBCR from Sub Saharan Africa, where 64.9% of the patients were diagnosed at late stages, of which 18.4% were metastatic [23]. On the other hand, our findings also contrast with the 76–92% early stages reported among breast cancer patients from populations in Nordic countries, Australia and Canada [24].

In the MENA region, efforts in cancer control and breast cancer screening have been scaled up in the last years, yet the programmes remain largely opportunistic [25]. Alongside low mammography coverage estimates (24), there is evidence from some Gulf countries regarding low uptake and barriers to breast cancer early detection among Arabic women This contrasts with our findings from Oman, Bahrain and UAE, where early-stage breast cancers were above 60%; nevertheless, UAE had more than 20% of the cases without stage information.

In Northern Africa, Morocco has made significant investments in improving breast cancer detection and treatment [26], [27] in the last years; however, our data from the Rabat registry indicate that breast cancer stage distribution has not shifted when compared to previously reported 2006–2008 data [28]. For Casablanca, a transition to use TNM replacing former use of SEER [29] impedes assessing changes in breast cancer stage; however, hospital-based studies at one major Casablanca institution in 2004, 2009 and 2018 report increasing diagnosis of early-stage breast cancer over time [30], [31].

In the case of Turkey, where specialized centres for opportunistic and organized breast cancer screening exist in 81 provinces since 2008 [32], our results (SEER) indicated a predominance of localized cancers in Izmir, as opposed to Antalya where most cancers were diagnosed already with regional extension. Despite a relative low mammography uptake in the country the diverging results could reflect a better mammography uptake in the western region (where Izmir is located) [32], [33].

In a population-based analysis of breast cancer stage and mortality [21], 20 countries (of 148 evaluated) that achieved annual mortality reductions of 2% or greater (for at least 3 consecutive years since 1990) had met the target of having 60% or more of breast cancer patients diagnosed with early stage (I/I) disease. Moreover, some countries met these targets in the absence of population-based screening programmes, suggesting that a combination of improved breast cancer awareness and education at the community and the primary care/provider levels coupled with health systems strengthening can be a very effective means to increase access to early diagnosis and treatment and as such reduce breast cancer mortality.

A major strength in this study is the reporting of population- based data on breast cancer stage, as opposed to summary reviews performed in other regions (or including also this region), but predominantly based on hospital data [21], [34], [35]. Despite the great value of hospital- based series, there is a danger to generalize such data for a country, as they provide only a skewed representation of the population getting the diagnosis and treatment in tertiary centres. However, it is also important to highlight that our findings might not be representative for the MENA region, as some countries still do not have an operational PBCR, and among the participating registries, some questions also had a very low response rate. In addition, quality and timeliness of data is a concern. Certainly, some results like the lack of stage IV cancers in Bahrain and Batna or the timeliness for stage data submitted by two registries (data with more than 10 years of delay), merit further exploration. Some of the findings regarding timeliness, data quality and reporting also point out the need of having and maintaining high-profile staff to analyse, disseminate and translate the information to the relevant stakeholders.

IARC, GICR and the UICC have put in place multiple efforts to improve stage data and facilitate transitioning towards use of TNM globally. Essential TNM was developed as a complement to TNM when information on T, N, M is missing in clinical records [5]. The guidelines, focusing initially on breast, cervix, colorectal and prostate cancer, have been recently translated to French (available at https://gicr.iarc.fr/) and Turkish, with Arabic translation planned to ensure wider use in the MENA region. In addition, IARC and related partners recently launched CanStaging + , an online tool for TNM staging that is open source and available for use both for clinical and public health community [36].

## 5 Conclusions

Cancer stage data at the population level are critical for planning evaluation and monitoring of cancer control. Even if the overall proportion of early-stage breast cancer in MENA is above the threshold specified by the WHO Global Breast Cancer Initiative, there are large differences across the region as well as within the countries, reflecting underlying inequalities. The favourable results of early cancer (TNM I + II) in two thirds of breast cancer patients reported in the present study need to be interpreted with caution and followed up in time. Improved cancer surveillance, including TNM stage data, will help tailor the different cancer control strategies and monitor their impact. In resource limited settings, restraining the collection of stage information only to cancers amenable to early detection, such as, breast, cervix and colorectal cancers, could contribute to improving quality, completeness and attain better dissemination.

## CRediT authorship contribution statement

**Marion Piñeros**: Conceptualization, Methodology, Data curation, Analysis, Writing − original draft preparation, Writing − review & editing, Visualization. **Ophira Ginsburg:** Writing − review & editing. **Karima Bendahhou**: Writing − review & editing. **Sultan Eser**: Writing − review & editing. **Wael A Shelpai**: Writing − review & editing. **Heba Fouad**: Writing − review & editing. **Ariana Znaor:** Conceptualization, Methodology, Data curation, Analysis, Writing − review & editing, Supervision, Visualization. **Staging Survey Group:** Investigation, Review.

## Declaration of interest

None; the authors declare no competing interests.
