# Supplementary material for: Staging practices and breast cancer stage among population-based registries in the MENA region
Source: Cancer Epidemiol. 2022 Dec;81:102250. doi: 10.1016/j.canep.2022.102250 (PMC9693698; doi:10.1016/j.canep.2022.102250)

**Supplementary Figure 1. Stage at diagnosis (SEER classification) among breast cancer patients; selected PBCR in the MENA region**


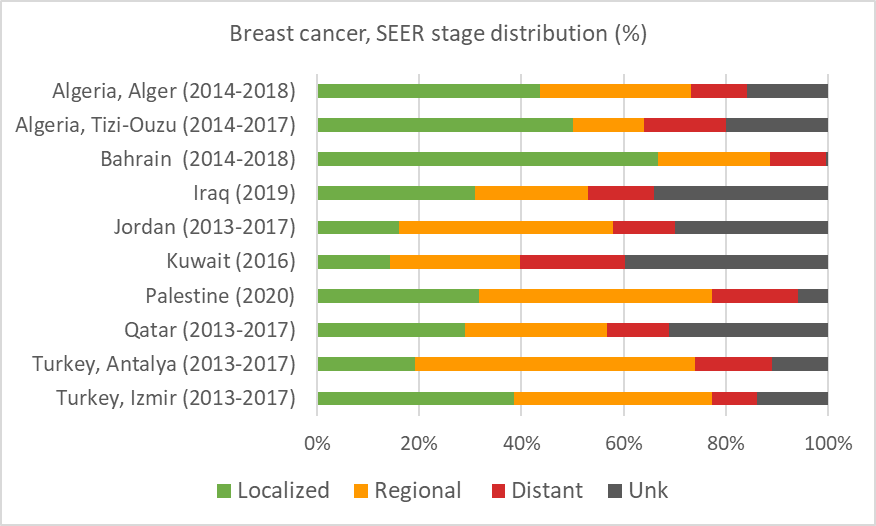

Supplement: Supplementary file 1 — Supplementary material. [file mmc1.docx]
